# Left Atrial Function as Assessed by Speckle-Tracking Echocardiography in Hypertension

**DOI:** 10.1097/MD.0000000000000526

**Published:** 2015-02-13

**Authors:** Ting-Yan Xu, Jing P. Sun, Alex Pui-Wai Lee, Xing S. Yang, Ling Ji, Zhihua Zhang, Yan Li, Cheuk-Man Yu, Ji-Guang Wang

**Affiliations:** From the Center for Cardiovascular Evaluations, The Shanghai Institute of Hypertension, Ruijin Hospital, Shanghai Jiaotong University School of Medicine, Shanghai (T-YX, YL, J-GW); and Division of Cardiology, S.H. Ho Cardiovascular and Stroke Centre, Prince of Wales Hospital, Chinese University of Hong Kong, Hong Kong, China (JPS, AP-WL, XSY, LJ, ZZ, C-MY).

## Abstract

We investigated left atrial (LA) function in relation to hypertension using 2-dimensional speckle-tracking echocardiography (STE) in subjects with preserved left ventricular (LV) ejection fraction, while accounting for LA enlargement and LV mass and diastolic function.

We performed standard 2-dimensional and Doppler echocardiography and LA volumetric measurements and STE strain imaging in hypertensive patients (systolic/diastolic blood pressure ≥140/90* *mmHg, or use of antihypertensive drugs, n = 124) and age- and sex-matched normotensive subjects (n = 124). We measured the peak LA velocity, strain, and strain rate during systole and early and late diastole, respectively. We investigated the associations of interests in the presence or absence of LA enlargement (LA volume index ≥28 mL/m^2^).

Hypertensive and normotensive subjects had similar LV ejection fraction and LA diameter (*P *≥* *0.22). However, hypertensive compared with normotensive subjects had enlarged LV and impaired diastolic function, and had increased LA volumetric measurements and decreased LA emptying fractions (*P* < 0.0001). Hypertensive patients also had impaired LA function, as measured by STE velocity, strain, and strain rate in general and in the absence of LA enlargement (*P* < 0.0001). The differences in LA STE strain rate during LV systole and LA contraction between hypertension and normotension in the absence of LA enlargement remained statistically significant (*P *< 0.001), after adjustment for age, sex, and LV mass index and E/E’.

Hypertension is associated with impaired LA function, as assessed by STE strain imaging technique, even before LA enlargement develops and after LV remodeling is accounted for.

## INTRODUCTION

Normal left atrial (LA) structure and function is essential for the cardiac function.^1^ Indeed, in addition to LA enlargement, LA functional abnormalities may also predict the occurrence of atrial fibrillation.^2,3^ Recent studies suggest that LA structural remodeling and/or functional impairment might play a part in the pathogenesis and development of ventricular disorders, such as heart failure with or without preserved ejection fraction.^4,5^ However, LA, especially its function, is insufficiently studied because of measurement difficulties. Standard echocardiography only routinely measures the LA diameter. Tissue Doppler imaging has limited accuracy of measurement and hence restricted use in clinical and even research settings.^6^

Speckle-tracking echocardiography (STE) makes it possible to measure LA function with relatively high accuracy.^7^ More importantly, the STE strain imaging technique allows detection of LA functional impairment at the very early stage, such as in the absence of LA enlargement. We recently performed STE measurements in a series of hypertensive and normotensive subjects. In the present study, we compared hypertensive with normotensive subjects in the prevalence of LA enlargement and functional impairment as assessed by the volumetric measurement and deformation indexes, and investigated the association of LA functional impairment with LA enlargement, while accounting for left ventricular (LV) structural remodeling and diastolic dysfunction in these hypertensive and normotensive subjects.

## MATERIALS AND METHODS

### Study Population

From September to November 2013, we enrolled 124 consecutive primary hypertensive patients from a population cohort (n = 100)^8^ and from our specialized hypertension clinic (n = 24). Of them, 26 had previously established hypertension, and 98 had newly diagnosed hypertension. Hypertension was diagnosed as a blood pressure of ≥140/90 mmHg or as the current use of antihypertensive drugs. Patients were excluded if they had atrial fibrillation, significant valvular heart disease, or poor imaging quality. As a control group, we recruited 124 sex- and age-matched subjects without hypertension from the apparently healthy hospital staff (n = 88) and from the above-mentioned population cohort (n = 36).^8^ In addition, these healthy individuals were without any history of cardiovascular disease and risk factor and were normal in physical examinations. The study protocol was approved by the Ethics Committee of Ruijin Hospital, Shanghai Jiaotong University School of Medicine, China. All subjects gave written informed consent.

### Standard Echocardiography

Comprehensive transthoracic echocardiography was performed using a cardiac ultrasound system with digital storage capacity (E9, General Electric, Milwaukee, WI, USA). LA diameters, LV diastolic diameters (LVDD), thickness of the interventricular septum (IVS) and thickness of the LV posterior wall (LVPW) were measured according to the American Society of Echocardiography guidelines.^9^ Conventional parasternal short axis views at basal, middle, and apical levels and apical 4-chamber, 2-chamber, and 3-chamber views were obtained. Three consecutive cardiac cycles in sinus rhythm were digitally stored for subsequent analyses. All the images were obtained at a frame rate of 60–90 frames/s. LV mass in grams was calculated from M-mode echocardiograms according to the formula LV mass = 0.8 × (1.04 × [{LVDD + LVPW + IVS}^3^ − {LVDD}^3^]) + 0.6, described by Devereux et al.^10^ LV mass was indexed to body surface area as LV mass index in gram per meter square. LV hypertrophy was defined as a LV mass index of at least 115 g/m^2^ and 95 g/m^2^ for men and women, respectively.^11^ The LV volumes were measured by 2D Simpson method.

The peak early (E) and late (A) transmitral flow velocities, the ratio of early-to-late peak velocities, and the deceleration time of E velocity were measured. The mitral flow velocity was obtained from apical 4-chamber view by placing a pulsed-wave Doppler sample volume between mitral leaflet tips during diastole. The curve of continuous wave Doppler was put between the LV outflow and inflow tracts and recorded a trace of LV outflow and inflow waves from the apical 5-chamber view.

Pulmonary vein Doppler signals were acquired in the apical 4-chamber view by interrogating the right upper pulmonary vein. Color flow imaging was used to align the beam parallel to pulmonary vein flow. A sample volume was placed 1 cm into the pulmonary vein. Pulmonary venous Doppler systolic and diastolic peak velocities and pulmonary vein atrial reversal wave were obtained.

Tissue Doppler imaging was performed to measure myocardial velocities. Pulsed sample volume was placed at the septal corner of mitral annulus; early diastolic (E’) and late diastolic (A’) myocardial velocities were recorded. The E/E′ ratio was then calculated.^12^

### Volumetric Measurements of LA

The LA area was measured with a planimetry for 4-chamber and 2-chamber views by tracing the endocardial border, excluding the confluence of the pulmonary veins and the LA appendage. LA volume was measured at end-systole, before P-wave, and end-diastole, respectively.^13^ Maximum LA volume (LAVmax, measured on the 2D frame just before mitral valves opening), pre-atrial contraction LA volume (LAVpreA, measured on the frame just before the onset of atrial emptying), and minimum LA volume (LAVmin, the smallest LA volume measured on the frame at end-diastole) were computed separately according to the American Society of Echocardiography guidelines using biplane modified Simpson method of discs.^9^ The indexes and formulas calculated from the volumes were as follows: the total LA emptying fraction = (LAVmax − LAVmin)/LAVmax × 100%; the active LA emptying fraction =  (LAVpreA − LAVmin)/LAVpreA × 100%; and the passive LA emptying fraction =  (LAVmax − LAVpreA)/LAVmax × 100%. The LA size was represented by LA maximal volume measured at end-systole and indexed by body surface area (LA volume index). Measurements were repeated 3 times in each individual, and the average was used for analysis. We defined LA enlargement as a LA volume index of ≥28 mL/m^2^.^9,14^ The accuracy of this echocardiographic technique has been validated previously against the computed tomography and magnetic resonance imaging methods.^15,16^

### Measurements of LA Deformation by STE

The LA endocardial border was manually defined using a point-and-click technique. An epicardial surface tracing was automatically generated by the system, creating a region of interest, which was manually adjusted to cover the full thickness of the myocardium in the systolic frame. Before processing, a cine loop preview was used to confirm whether the internal line of the region of interest followed the LA endocardial border throughout the cardiac cycle. The software divided the region of interest into 6 segments and generated the averages of the values and curves of velocity, strain, and strain rate for each segment. Segments with suboptimal image were rejected by the software and excluded from the analysis. For the estimation of the LA segmental function, each atrial wall was divided into 2 segments (upper and lower) from apical 4-, 2-, and 3-chamber views. The LA roof segments in each view were excluded in this study due to the discontinuity of LA wall as a result of the connection to the pulmonary veins.

**FIGURE 1:**
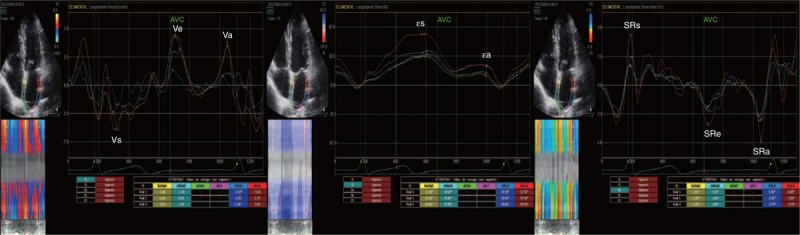
Speckle-tracking echocardiographic strain imaging technique. The left atrial (LA) strain (ε) was calculated with R-wave onset of the electrocardiogram as a reference point, which enabled the recognition of the first peak ε (εs), second peak ε (εa), and the difference between the first and second peaks (εe = εs–εa), corresponding to LA reservoir, contractile and conduit functions, respectively (middle). For the LA strain rate (SR) and velocity (V), the positive peak strain rate (SRs) and negative peak velocity (Vs) at the beginning of LV systole, the first negative peak strain rate (SRe) and positive peak velocity (Ve) at the beginning of LV diastole, and the second negative peak strain rate (SRa) and positive peak velocity (Va) during LA contraction were identified corresponding to the LA reservoir, conduit, and contractile functions, respectively (right and left).

The LA STE curves were obtained using R-wave onset of the electrocardiogram as a reference point, which enabled the recognition of first peak ε (εs), second peak ε (εa), and the difference of the first and second peaks (εe = εs–εa), each corresponding to LA reservoir, contractile, and conduit functions, respectively. The LA strain rates and velocities were measured from corresponding curves, respectively, as the positive peak strain rate (SRs) and negative peak velocity (Vs) during LV systole, the first negative peak strain rate (SRe) and positive peak velocity (Ve) during LV early diastole, and the second negative peak strain rate (SRa) and positive peak velocity (Va) during atrial contraction. Figure 1 shows the curves of LA strain, strain rates, and velocities. Because there is no agreed threshold for the definition of abnormal LA function as assessed by strain rate, we defined abnormal LA function as SRs ≤1.4 L/s, SRe ≤1.0 L/s, or SRa ≤1.6 L/s, which is the lower boundary of the 95% confidence interval in 121 healthy subjects in a previous publication.^17^

### Intra- and Interobserver Variability

The intra- and interobserver variability was assessed in 40 randomly selected subjects, and calculated as the standard deviation divided by the mean of the intraobserver, intersession, or the inter-observer differences. To assess the intraobserver variability, randomly selected images were analyzed at a different time by an observer blinded to the results of previous measurements. To assess the interobserver variability, randomly selected images were analyzed by a second observer blinded to the values obtained by the first observer. The intraobserver variability was 9.7%, 9.7%, and 9.1%, respectively, for LA Vs, Ve, and Va and 7.2%, 8.6%, and 9.7%, respectively, for LA εs, εe, and εa. The corresponding values for the interobserver variability were 8.5%, 9.0%, and 8.7% and 6.9%, 8.4%, and 9.7%, respectively.

### Statistical Methods

For database management and statistical analyses, we used the SAS software, version 9.1.3 (SAS Institute Inc, Cary, NC, USA). Means and proportions were compared with the Student *t* test and Fisher exact test. We performed single and multiple regressions to study associations of interest. We studied statistical interaction between 2 variables by including the product of these 2 variables in the model.

## RESULTS

### Characteristics of the Study Participants

The study population included 124 hypertensive patients and 124 normotensive subjects. They had similar (*P* ≥ 0.19) sex distributions (45.9% of men) and mean (±SD) age (52.7 ± 11.5 years). Hypertensive patients, compared with normotensive subjects, had significantly (*P* < 0.0001) higher systolic/diastolic blood pressure (149.5/90.1 vs 118.8/74.8 mmHg, Table 1). Of the 124 hypertensive patients, 14 (11.2%) took antihypertensive medication.

**TABLE 1:**
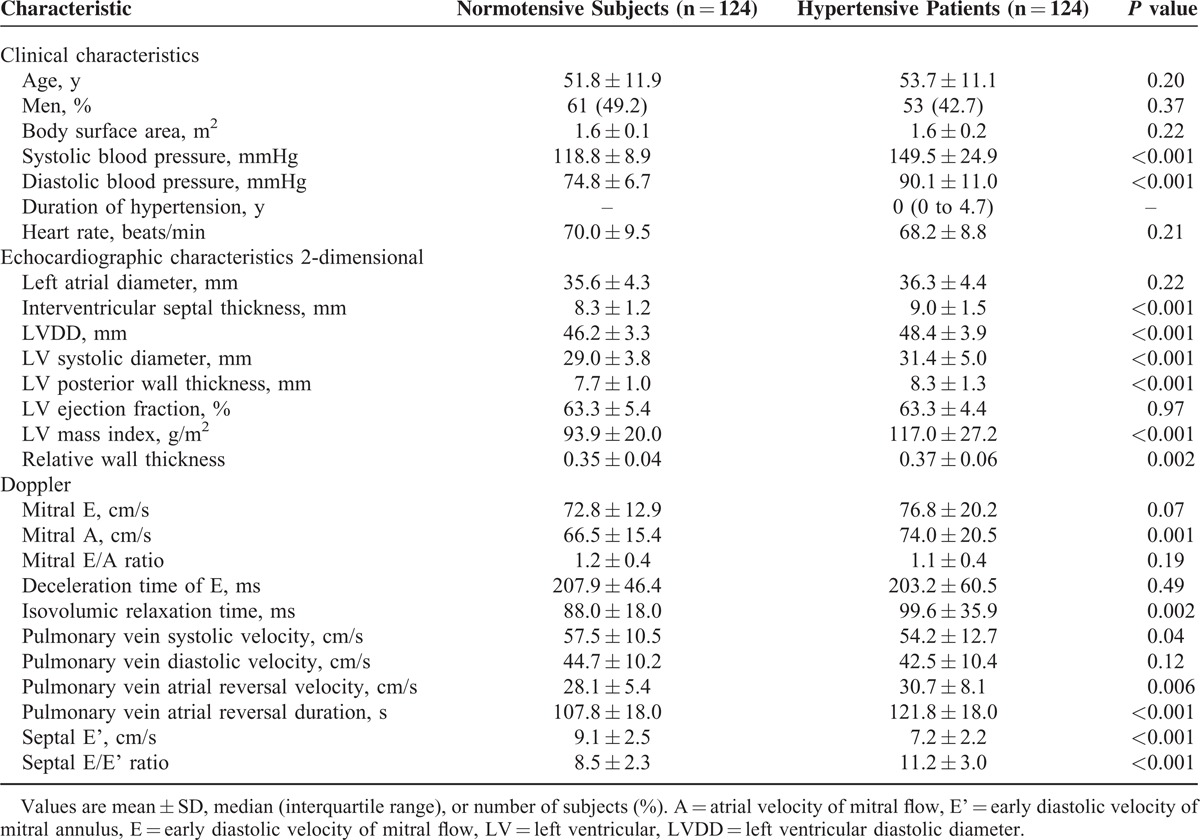
Clinical and Echocardiographic Characteristics of Hypertensive and Normotensive Subjects

Hypertensive patients and normotensive subjects had similar (*P* ≥ 0.22) LA diameters (36.0 ± 4.3 mm) and LV ejection fraction (63.3% ± 4.7%, range 51.0%–75.5%) as assessed by standard echocardiography. However, hypertensive patients, compared with normotensive subjects, had enlarged LV and poorer diastolic function, as exemplified by greater LV wall thickness, LVDD, LVDS, and LV mass index (*P* ≤ 0.002) and by longer isovolumic relaxation time, increased pulmonary vein atrial reversal velocity and duration, lower pulmonary vein systolic velocity and E’ and higher E/E’, respectively (*P* ≤ 0.04, Table 1).

### Association of LA Volumetric Measurements and Deformation Indexes with Hypertension

Hypertensive patients, compared with normotensive subjects, had enlarged LA volumes and decreased LA emptying fractions, as measured by LA Vmax, VpreA, and Vmin (*P* < 0.001) and by total, passive, and active emptying fractions, respectively (*P* ≤ 0.006, Table 2). The prevalence of LA enlargement was 49.4% (n = 60) and 6.5% (n = 8) in hypertensive patients and normotensive subjects, respectively (*P* < 0.0001).

**TABLE 2:**
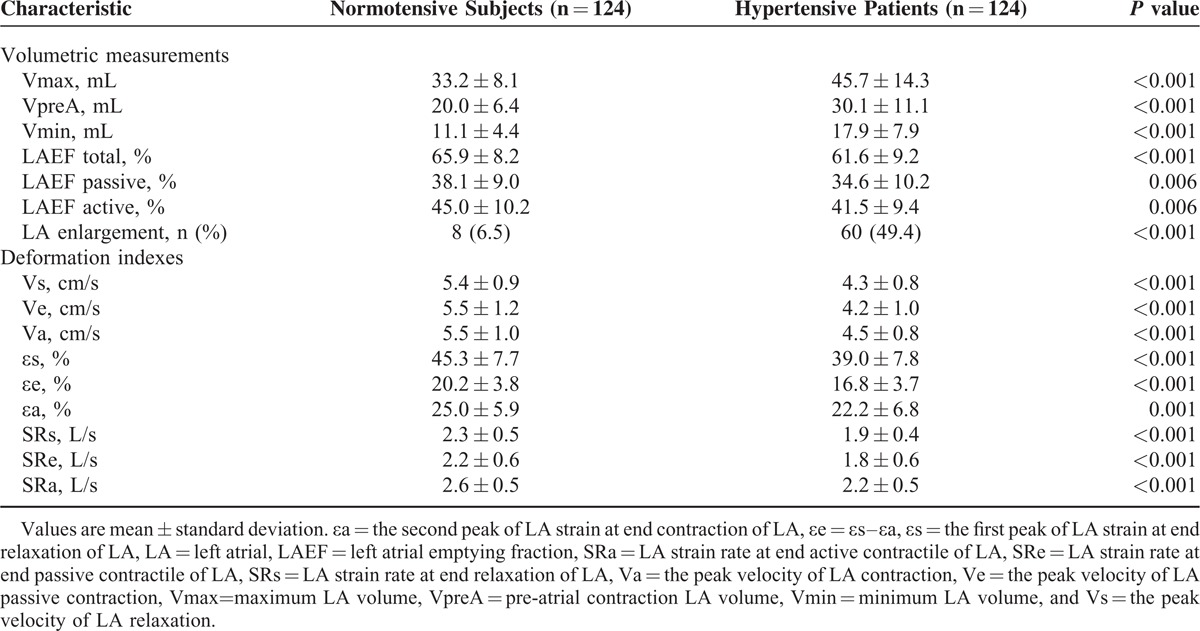
Comparison of Left Atrial Volumetric Measurements and Deformation Indexes Between Hypertensive and Normotensive Subjects

**FIGURE 2:**
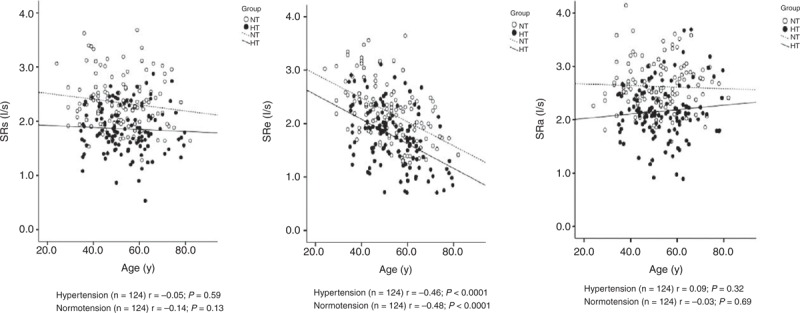
Scatter plots on left atrial strain rates during left ventricular systole (SRs, left), early diastole (SRe, middle), and left atrial contraction (SRa, right) according to age and the presence (dot) or absence (circle) of hypertension. Regression line was drawn for hypertensive patients (full line) and normotensive subjects (dashed line) separately. Correlation coefficient (*r*) and its *P* value are given.

Hypertensive patients, compared with normotensive subjects, also had decreased LA deformation indexes, as measured by LA velocity, strain, and strain rate during LV systole, early diastolic, and LA contraction (*P* ≤ 0.001, Table 2). These differences between hypertension and normotension were similar across the whole age range from 20 to 80 years, despite that SRe decreased significantly with age (*P* < 0.0001, Figure 2). The prevalence of abnormal LA function was 14.5% (n = 18) and 1.6% (n = 2) in hypertensive patients and normotensive subjects, respectively (*P* < 0.0001).

### Association of LA Functional Impairment With Structural Enlargement

**FIGURE 3:**
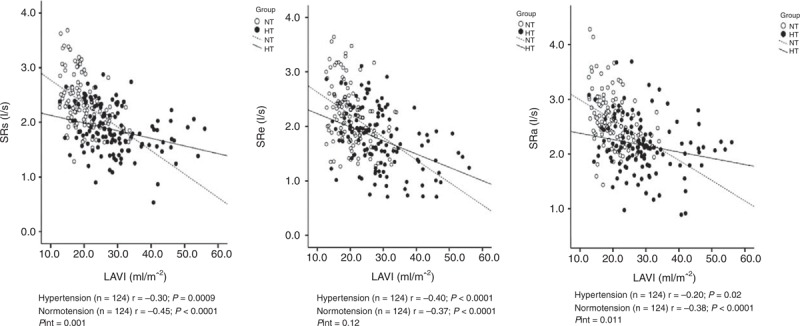
Scatter plots on left atrial strain rates during left ventricular systole (SRs, left), early diastole (SRe, middle), and left atrial contraction (SRa, right) according to left atrial volume index and the presence (dot) or absence (circle) of hypertension. Regression line was drawn for hypertensive patients (full line) and normotensive subjects (dashed line) separately. Correlation coefficient (*r*) and its *P* value are given. The *P* value for the interaction between left atrial volume index and hypertension in relation to left atrial function is also given (*P*int).

The LA deformation indexes significantly (*P* < 0.0001) decreased with LA enlargement in hypertensive patients as well as normotensive subjects. However, there was significant (*P *≤* *0.01) interaction between LA volume index and the presence or absence of hypertension in relation to the LA deformation indexes measured during LV systole and LA contraction. In the presence of hypertension, these LA deformation indexes significantly (*P* < 0.001) decreased, even when LA volume index remained in the normal range (Figure 3). In spite of nonsignificant interaction for LA deformation indexes measured during LV early diastole, hypertensive patients also had significantly decreased LA deformation indexes in the absence of LA enlargement (*P* < 0.0001). In the presence of LA enlargement, none of the differences between hypertensive patients (n = 60) and normotensive subjects (n = 8) reached statistical significance (*P* ≥ 0.08).

In multiple linear regression analyses adjusted for sex and age, we investigated contribution of hypertension to decreased LA deformation indexes in the absence of LA enlargement, while accounting for LV mass index and E/E’. Although LV mass index was significantly associated with all the LA deformation indexes (r = −0.17 to −0.26, *P *≤* *0.04), hypertension remained significantly associated with strain rate during LV systole and LA contraction (r = −0.29 for both, *P *≤* *0.001). If in the regression model, LV mass index was replaced with LV hypertrophy and duration of hypertension was additionally adjusted for, these associations between hypertension and strain rate did not change materially (*P *≤* *0.01).

## DISCUSSION

Our findings are 2-fold. First, hypertensive patients had high prevalence of LA enlargement, which was associated with impaired LA function, as measured by the STE strain imaging technique. Second, in the absence of LA structural enlargement, hypertension was still associated with LA functional impairment, especially during LV systole and LA contraction, even after accounting for LV remodeling and diastolic dysfunction.

Our finding on the impaired LA function as assessed by STE strain imaging during LV systole and LA contraction in the absence of LA enlargement and in the presence of hypertension extends previous observations on this topic.^18–21^ There is consensus that hypertension is associated with impaired LA function, regardless of the measurement techniques, such as the phasic/volumetric measurements, indexes of mitral and pulmonary vein flows, and tissue Doppler imaging or STE deformation indexes by strain imaging techniques.^22–24^ However, these previous studies often did not account for LA enlargement or LV structural and functional remodeling or attributed the observed LA functional impairment to these atrial and ventricular abnormalities. After accounting for these apparent cardiac abnormalities, we found that hypertension was still associated with LA functional impairment, suggesting that LA strain imaging is particularly sensitive in assessing LA function in hypertension. It is possible that these 2 dynamic measurements, respectively, in the heart and systemic circulation, though separated by LV, may share common or similar mechanisms of pathogeneses.^25^ The STE strain imaging therefore might be useful in the assessment of target organ damage and in the initiation of antihypertensive treatment on several conditions, such as white-coat or masked hypertension.^26^ In the presence of impaired LA function, even white-coat or masked hypertension might be treated with hypertensive drugs.

Using multiple echocardiographic techniques, we found that hypertensive patients with mildly elevated blood pressure might have multiple structural and functional LA and LV abnormalities. Among others, LA enlargement had high prevalence and was closely related to LA and LV functions. This confirmatory finding might be clinically relevant. Current standard echocardiography only measures LA diameter, and can only unveil abnormalities at a late and probably irreversible stage. In hypertension, a more thorough echocardiographic evaluation of LA may be necessary and useful in the choice of antihypertensive drugs and target blood pressure. There is some evidence that inhibitors of the renin-angiotensin system may provide more protection against LA diseases, such as atrial fibrillation.^27^

Our observation on the close association between LA enlargement and functional impairment is in line with the results of numerous previous studies involving various measuring techniques.^1^ This consistency to some extent validates the STE strain imaging technique in general and our measurements in particular. This 2-dimensional STE strain imaging technique allows simple and rapid evaluation of 3 phases of LA function, and may be used in the clinical setting. With a relatively short term of training, an echocardiographer, experienced in standard echocardiography, can manage in operating the technique with an acceptable intra- and interobserver variability between repeated measurements.

Our study should be interpreted within the context of its limitations. First, our study population was not a random sample. Although hypertensive and normotensive patients were well-matched in sex and age, selection bias is still possible. Second, hypertension is a major risk factor of coronary artery disease. Myocardial ischemia may contribute to the difference between hypertension and normotension. However, LV ejection fraction was normal in all subjects and similar between hypertension and normotension. Diastolic function had been accounted for in statistical analyses. Third, our study is cross-sectional and noninterventional, and hence, does not allow any inference of causality or reversibility. Finally, the sample size of our study is relatively small. One of the major limiting factors is that the STE analysis is at present still quite time-consuming.

## CONCLUSION

Hypertension is associated with impaired LA function, as assessed by STE strain imaging technique, even before LA enlargement develops and after LV structural and functional remodeling is accounted for. A major clinical implication of our finding is that STE strain imaging might be required to detect early impairment of LA function in hypertension, especially in the absence of LA and LV abnormalities on standard echocardiography. Nonetheless, because the STE analysis needs high-quality resolutions and has relatively high intra- and interobserver variability, this technique would require developed probes and software for future routine use in the clinical setting.
